# Application of Educational Psychology-Based Dance Therapy in College Students’ Life Education

**DOI:** 10.3389/fpsyg.2022.784568

**Published:** 2022-03-21

**Authors:** Haiyan Zhong, Chunhui Zhao, Fengrui Zhang, Ruizhi Zhang

**Affiliations:** ^1^College of Marxism, Northeast Agricultural University, Harbin, China; ^2^College of the Arts, Agricultural University of Hebei, Baoding, China; ^3^College of Life Science, Sichuan Agricultural University, Ya’an, China; ^4^College of Music and Dance, Hunan First Normal University, Changsha, China

**Keywords:** educational psychology, life education, dance therapy, college students, data analysis

## Abstract

The purpose is to strengthen the life education of contemporary college students and give better play to the vital role of life education in preventing college students’ mental diseases. Specifically, it discusses the role of dance therapy (DT) in College Students’ Life Education (CSLE). Firstly, based on educational psychology (EP), this manuscript analyzes the relevant theoretical concepts of EP and life education and discusses the importance of life education to contemporary college students. Secondly, following a Questionnaire Survey (QS) and using deep learning (DL) Convolutional Neural Network (CNN) and Facial Emotion Recognition (FER), this manuscript reviews and examines the CSLE’s current situation and the DT effect. Research findings are summarized combined with the QS results and scores of 20 subjects before and after five activities in 3 months. (I) After DT intervention, the positive dimensions of college students’ life values have improved, especially self-development and dedication, and their quality of life is refined. Thus, DT group counseling proves the positive role of DT in CSLE. (II) After DT intervention, 96.5% of the members think DT is effective. Therefore, EP-based DT is more effective and scientific in CSLE. The research findings provide a DT-based teaching concept for CSLE, explore the feasibility and effectiveness of life education, and enrich the DT scheme of CSLE. The research provides a practical reference for further applying DT in college students’ psychological education.

## Introduction

The present work aims to seek more diversified ways to prevent mental diseases by introducing the concept of dance therapy (DT). With the development of the Internet, the psychological problems of college students have attracted extensive attention, and the research on college students’ mental diseases has also increased. At present, the leading education on preventing college students’ mental diseases is centered on life education. Since the 21st century, more and more studies have shown that physical exercise will positively impact people’s psychology. Therefore, this manuscript will study its role in College Students’ Life Education (CSLE) combined with dance teaching. As a combination of Ideological and Political Education (IAPE) and psychology, life education has attracted the attention of scholars all over the world and drew the thinking and research of IAPE educators. Life education is a relatively new concept in China, and the life education system model with Chinese cultural characteristics is still in its infancy. As part of IAPE, the importance of life education can never be overemphasized. College students, in particular, are the pillar of China’s socio-economic development. Thus, there is a rising demand to cultivate college students with reliable and effective life education, help them explore the meaning of lives, realize life values, and lay a foundation for future study and life. At the same time, to make educators and students have a better experience in life education, it is very effective to integrate educational psychology (EP) into the whole teaching process (Allen and Lavender-Stott, 2020; Brotherson and Hoffman, 2020).

Dance therapy is a discipline and has become an industry as well. It has originated in Europe and risen in the United States. Its basic principle is to emphasize internal expression and publicize personality. The earliest practitioners of DT are dancers, who summarize their experience in dance career into their unique dance therapy methods. Traditional DT uses dance or improvization to treat social, emotional, cognitive, and physical disorders, enhance personal consciousness, and improve people’s minds. With the development of psychology, some dancers begin to introduce these methods and dance movement analysis into dance and psychology research (Yamada and Kawano, 2021). Particularly, DT based on psychological concepts can greatly impact people. Since the rise and popularization of DT, it has been combined with various disciplines and developed many new methods and research results. Internationally, DT has been applied to personal development, mental health counseling, and mental disease prevention. China has just introduced the concept of DT and is still seeking the initial stage of development, but most cases are used as the experience of psychological prevention (Candelieri, 2020).

Because people’s emotions are related to their views on life, this manuscript uses Emotion Recognition Technology (EMT) based on Deep Learning (DL) to represent the results of DT + life education. The key point of Artificial Intelligence (AI) ‘s Perceptual Ability (PA) lies in emotion perception. Only when machines truly understand emotion can they respond correctly to the environment in a human way of thinking. The human emotional state is extremely rich. Significantly, a machine needs to recognize various signals corresponding to multiple emotions to understand human emotions. The typical application of EMT is psychotherapy, in which psychologists can master the patient’s psychological state and help detect depression by judging the patient’s feelings. Of course, the role of EMT is not only reflected in psychotherapy but also plays an essential role in evaluating the advertising effect through the audience’s response, character analysis in the video, emotional robot, and medical rehabilitation of patients, among many other scenes. DL belongs to the broader category of Machine Learning (ML). DL can learn the high-level abstract data representation and automatically extract data features. Convolutional Neural Network (CNN) is one of the representative DL algorithms. It can well establish the relationship between pixels according to the data features of image mode, extract the semantic information in the image, and realize the image understanding.

From the perspective of EP, this manuscript analyzes the role and influence of DT in contemporary CSLE and discusses the problems and reasons existing in the process of CSLE. On this basis, a practical and effective DT scheme for CSLE based on EP is proposed. Then, the CNN is introduced to characterize the effect of DT through Facial Emotion Recognition (FER) technology, which provides a new idea and research direction for CSLE. This manuscript will study the problems existing in the current college students receiving DT. Following the Questionnaire Survey (QS) and feedback of the DT group, it proves that DT can significantly improve the effectiveness of CSLE. Finally, the experimental findings corroborate that DT can enhance students’ understanding of the goal, improve students’ life consciousness, and improve students’ interpersonal relationships. This manuscript creatively discusses the role of DT in CSLE from the perspective of EP, provides a more popular way for college students’ mental disease prevention education, and improves college students’ acceptance of life education. The DL technology will be applied to FER after DT treatment and provide technical support for the DT results and reference for the academic research of DL technology in the DT + life education field.

## Overview and Research Methods of Educational Psychology, Dance Therapy, and Life Education

### Overview of Educational Psychology

Educational psychology is a branch of psychology that specializes in the psychological laws of teaching and learning in the educational environment. Meanwhile, EP is a compulsory course for ordinary students, teacher-training, and psychology and pedagogy majors. With the advancement of the times, EP is extending to the teaching process of numerous majors. In this process, EP is used to study students’ and educators’ psychological phenomena and laws, promote the reform of education and teaching, and improve teaching quality, thus playing a positive role in the education and training of students (Burchell, 2021).

Educational psychology is around the interactive and systematic teaching and learning process, including five factors: students, educators, teaching content, teaching media, and teaching environment. It encompasses the nature of pedagogy and psychology. Applied EP and the development of modern biology, sociology, and psychiatry have a mutual influence on education. The contents of EP are constantly updated to meet social development needs.

To analyze and research EP, people often use observation, natural experiment, investigation, and interview methods to deeply explore the role of description, interpretation, and predictive control in educational practice. The research process has found some defects in pedagogy. These deficiencies are supplemented and improved through the study of EP (Gehlbach and Robinson, 2021).

### Overview of College Students’ Life Education

Life education is an argument about death education originated in the United States, also called death education. Its theoretical founder believes that learning life is learning death. Life education can help people realize the limitation and infinity of life, achieve the unity of subject and object, and discover the transcendence and sublimation of life. Through life education research, the content of CSLE can be extended, which is a practical form of CSLE (Clynes et al., 2020).

**FIGURE 1:**
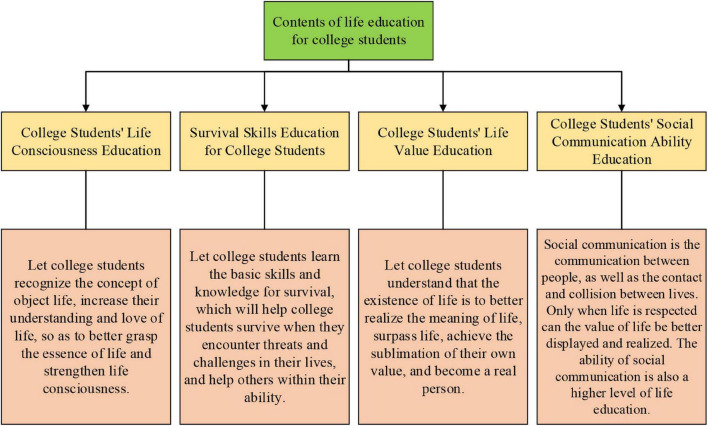
Contents of CSLE.

College Students’ Life Education is a part of IAPE in Chinese higher institutions. IAPE expects to revise college students’ negative outlook on life, guide them to respect lives, understand the meaning of life, find the value and significance of life, and improve their life quality (Aziz et al., 2020). Figure 1 shows the content of CSLE (Wei et al., 2021).

### Overview of Dance Therapy

**FIGURE 2:**
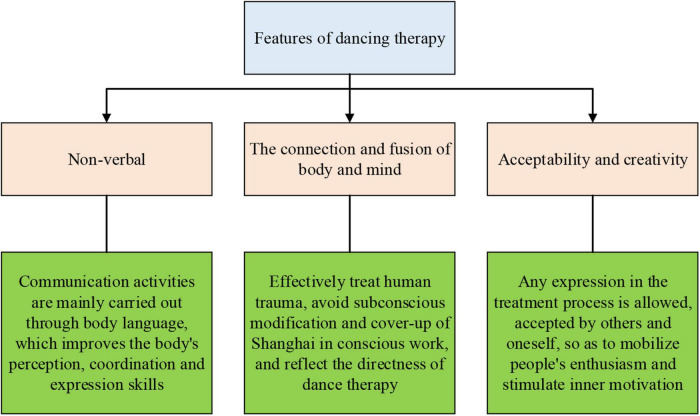
Features of DT.

Dance therapy is also known as dance movement therapy (DMT), which uses body movements as a medium to integrate and treat personal emotional and psychophysiological problems. DT is the combination of modern dance and psychology. It is a direct intervention to the individual subconscious through non-verbal communication, which assists the individual in the cognitive emotion of body and consciousness. DT does not involve learning or training dance skills but focuses on the movement’s dynamic process, expression, and meaning. DT contends that the body reflects the individual’s psychological state and recognizes self-existence. Meanwhile, DT is an essential cognitive component of psychodynamics in psychoanalysis. It expresses the correlation, interaction, and influence between body and consciousness linked through energy flow (Gail and Michal, 2020). Figure 2 displays the features of DT (Koch et al., 2019).

**FIGURE 3:**
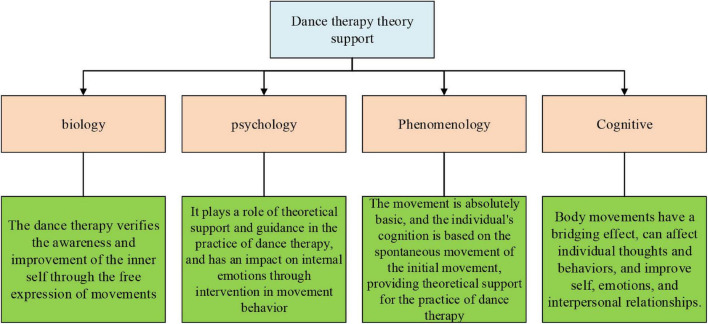
Theoretical support for DT.

Figure 3 outlines several academic supports obtained during DT (Stănescu and Tomescu, 2021).

Since the introduction of DT into China, it has seen wide applications in many directions. At the same time, a relatively complete theoretical DT system has formed outside China. In China, the content of DT has more similarities and conjunctions points with that of life education. With DT, Chinese CSLE can develop reasonably and effectively (Ko, 2020).

This manuscript mainly researches CSLE through a combination of EP and DT. There are few studies on the integration of EP and DT in CSLE. With the deepening of interdisciplinary science, more studies have been on the fusion of DT and psychology in recent years. For example, some studies have combined DT with psychology to study the depression of middle school students and college students. Some scholars have also combined DT with EP to examine college students’ self-efficacy. This manuscript combines the concept of EP with DT to study the CSLE. The research method mainly involves the QS method.

## Research Method

### Emotion Recognition Based on Dance Therapy Technology

There are many ways to express human emotion, leading to various ER patterns. Previous related research mainly focused on Image-based FER and Human Posture Recognition (HPR), text-based ER, speech-based ER, physiological signal-based ER, and multimodal ER. In traditional ER methods, Feature Extraction (FE)’s preprocessing process includes: 1, FE; 2, feature dimensionality reduction; 3, feature selection. In particular, FE methods encompass static image-based FE and dynamic image-based FE.

Deep learning technology has the superior ability of FE and classification. CNN is one of the classical networks in DL technology, and its image processing (IP) performance is outstanding. Therefore, this manuscript mainly studies the image-based ER combined with DL technology. This section uses 26 discrete categories and three continuous dimensions to build the model (Abdullah et al., 2021). Due to the structural shortcomings of DL, this section introduces DenseNetHE and Xception for network optimization. Dense Xception consists of an FE network and a feature fusion network. The FE network includes three sub-networks to extract the features of face, action, and context, respectively. Feature fusion network uses fully connected fusion three-way features to predict 26 discrete categories and three continuous dimensions. Each subnetwork is composed of five Dense Xception blocks. Firstly, the source image is deformed and convoluted to obtain the deformation invariant feature maps of 64 channels, and then these feature maps pass through five Dense Xception blocks in turn. In the middle of each block, it places a one × one convolution as the conversion layer to reduce the channel of the characteristic graph by half. Additionally, a two × two average pooling layer is placed after the first three blocks to retain more spatial information in the feature map. Each Dense Xception block is the basic module of FE. The original image information is the input of the deformed convolution layer, and its convolution kernel is 3 × 3. Meanwhile, the same object may show different sizes, attitudes, angle changes, or even non-rigid deformation in the image. The first three Dense Xception blocks are followed by one × one transfer floor and two × two average pooling. The Dense Xception block adopts the dense connection mode of the dense net. After the first three Dense Xception blocks, the average pooling can reduce the picture size from 64 × 64 to 8 × 8, dramatically reducing the amount of calculation and further improving the network’s generalization ability. The last two Dense Xception blocks only pass the transformation layer. At this stage, the feature map is small enough. Concerning enough space information, it does not use a pooling operation. Afterward, a two-way full connection operation reduces the superimposed three-way feature map to 26 and 3. The final features include two types: 26 and 3. Finally, the sigmoid function maps the discrete categories into [0,1] to predict each type.

The experimental samples are selected from the Emotions in Context (EMOTIC) data set, downloaded from http://sunai.uoc.edu/emotic/index.html. There are 23,554 images and 34,320 labeled samples, each framing people in the original image. There are 25% of images with no face presented in the data set, and the rest might have gotten people’s faces occluded. It is almost impossible to realize the FER in the past, but facial expression is one of the most direct and natural ways humans express emotion. Therefore, in this experiment, the facial expression is added as information input while combining context and action to use the face information in the data to obtain more comprehensive network information. Then, this section proposes FER through the combination of face, action, and context. Therefore, all samples’ face frames are manually marked to train the model, with 34,320 pieces marked in total.

### Questionnaire Survey and Experimental Methods

#### Literature Review

It analyzes and summarizes relevant theoretical knowledge and basis by querying the relevant literature of EP, life education, CSLE, and DT.

#### Experimental Method

It designs the activity plan of DT from the conjunction points of DT and CSLE, combined with the appropriate methods of DT.

#### QS Method

It analyzes college students’ life values and life quality through the QS design and statistical analysis. Life values are analyzed from six aspects: self-development, suffering acceptance, death acceptance, inaction, pleasure, and dedication, and the life quality is analyzed from the physiology, psychology, and social fields. The plotted data result can intuitively illustrate the impact of DT on college students’ life values and life quality (HyeRyeon et al., 2020).

Through the investigation and analysis of CSLE and life values, this manuscript discusses the situation of CSLE based on EP. Further, following the investigation and data analysis of college students’ feedback before and after the experimental activities, the existing EP-based DT life education problems are explored, and corresponding countermeasures and suggestions are put forward.

In this experiment, DT group members are recruited from a university. At the same time, according to the content of CSLE and combined with the relevant DT techniques, the group activity scheme of DT is designed. The range of the activity starts from the understanding of the object, allowing group members to temporarily shift their thinking and attention to the body itself. Then, it develops, adjusts, and connects various body parts, uses the body to express emotions and feel others’ feelings, improves members’ interpersonal relationship processing ability through training, and carries out intentional dance exercises to explore the meaning of life. Finally, the body and consciousness are integrated to achieve the goal of physical and mental harmony (Morris et al., 2021). Before the activity, the group members are pre-tested on life values and quality of life, followed by eight group activities of DT. After the activity, the DT group members are post-tested on life values and quality of life. Through the statistics and analysis of the pre-test and post-test data, this manuscript discusses whether DT is effective for CSLE and evaluates the subjective role of the activity through activity feedback form and interview.

This experiment will carry out ER twice before and after formal DT and compares the recognition results to provide technical support for presenting the effect of DT. The experiment conducts eight DT group activities from March 2021 to May 2021. The experimental group will carry out two activities every Saturday, each time for 1.5 h, and rested for half an hour, a total of 4 weeks and eight activities. QS, observation, and interview measure students’ emotional variations before and after the experiment, and the data are collected and counted. Finally, the data are analyzed by Statistical Package for the Social Sciences (SPSS) 19.0 to investigate the experimental effect. The subjects are interviewed, respectively, at the beginning, middle, and end of the experimental intervention. The subjects’ status is recorded and analyzed at each stage. Afterward, each activity’s impact on the subjects is compared to examine whether the subjects’ self-feeling has improved during DT. After the activity, the subjects are required to fill in the feedback form. The experiment compares and analyzes the experimental results from both quantitative and qualitative aspects. The satisfaction score in the QS in the Supplementary Appendix will only be used as one of the considerations in the section “Results.”

This experiment conducts a QS on group members before and after the intervention, respectively. CSLE mainly starts from two aspects: establishing college students’ life values and improving college students’ life quality. Therefore, the Life Values Scale and the Life Quality Scale are employed to measure group members (Millman et al., 2021). Afterward, the generic analysis is performed on the feedback form, open questions, and interview records of the group members and through observation and communication. Generic analysis refers to the process of finding recurring words and essential concepts that can explain these phenomena in data. In this process, data with the same attributes are classified into the same category and represented by a specific key name (Cao et al., 2021). The generic analysis of the collected materials can uncover the subjective tendency and role of DT group counseling in life education. Through the group discussion in the activity, the subjects carry out voluntary expression and complete communication of feelings after each activity. The variation of group members’ subjective feelings is integrated and analyzed after each activity by recording the communication content. After a full-scale activity, one-to-one interviews will be conducted to exchange ideas with the group members who have less communication or have special circumstances. The interview aims to evaluate the members’ participation and activity defects in the overall process and analyze whether the DT can improve the group members’ subjective cognition of life education.

In this group experiment, the subjects are recruited through the school network, new media, and the school advertising board. At the same time, to prevent too-small sample size from subjects’ loss, 32 volunteers are selected from the school, including one male and 31 female, which ensures the voluntarily of the subjects. Then, the experimental process and time are explained. A group contract is signed with everyone’s consent to reduce the subjects’ resistance, ensure experimental accuracy, and minimize the loss of subjects.

### Analysis of Educational Psychology, Dance Therapy, and Life Education

The ER results show that the subjects’ emotion has significantly improved, indicating that DT plays a positive role in CSLE.

### Analysis of Dance Therapy and College Students’ Life Education

The content analysis of the conjunction points of DT and CSLE is illustrated in Table 1.

**TABLE 1:** Conjunction points of DT and CSLE.

Conjunction level	Body level	Consciousness level	Interpersonal level
Content analysis	The core of CSLE is life (as opposed to death). The process of DT also reflects the recognition of life. DT techniques and activities enable the subjects to feel their bodies, express their consciousness through the body, feel the carrier of life, and connect their bodies and consciousness closer, thereby helping themselves better face life	CSLE includes the cultivation of college students’ love of life, positive outlook on life, and self-development. The process of DT is an intervention from body level to consciousness level, and it is also the unified and coordinated development of one’s own body and consciousness	CSLE focuses on the education of their interpersonal communication. Good interpersonal communication and sociability are essential components of CSLE. One aspect of DT is the cultivation of interpersonal relationships, which is reflected everywhere in the process of treatment

Table 1 shows the content analysis results of the conjunction points of DT and CSLE. Obviously, some specific topics of DT coincide with life education content for college students. Thus, DT can solve some problems in CSLE, and the life education implemented through DT can be more accessible and understandable for college students. DT can significantly promote CSLE in China (Chen, 2019; Sengupta and Banerjee, 2020).

Many in-depth studies have been conducted on interpersonal communication in DT and CSLE, finding that verbal information expression accounts for only 35% in terms of interpersonal communication. In comparison, non-verbal expression reaches 65%, and some experts have put forward the equation: information expression = 7% intonation + 38% voice + 55% body language. This proves the importance of body movement in communication. Therefore, life education implemented through DT can help college students adapt to collective activities by exercising and analyzing their limb movements (Swaine et al., 2020).

### Analysis of Dance Therapy in Life Education

**FIGURE 4:**
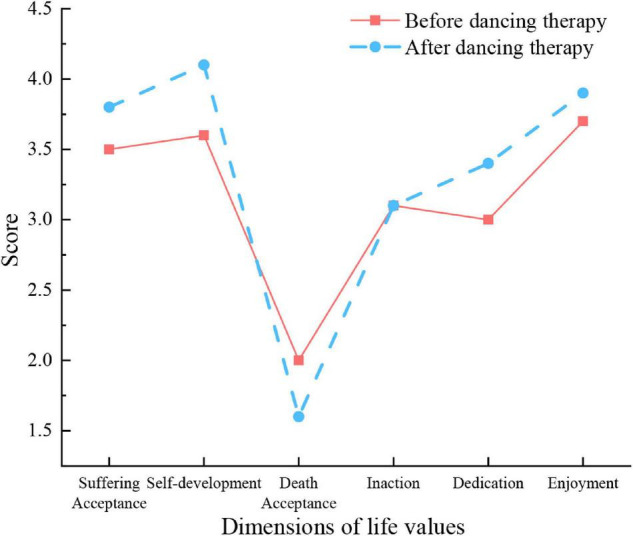
QS of college students’ life values before and after DT.

The scores of college students’ life values before and after DT are shown in Figure 4.

Figure 4 displays the scores of DT group activities in the relevant dimensions of college students’ life values. The data are the QS results and scores of a DT group with 20 subjects before and after five activities in 3 months. Apparently, after the DT, the positive dimensions of college students’ life values have been improved. The dimensions of self-development and dedication are more pronounced, proving the positive impact of DT on college students’ life values.

**FIGURE 5:**
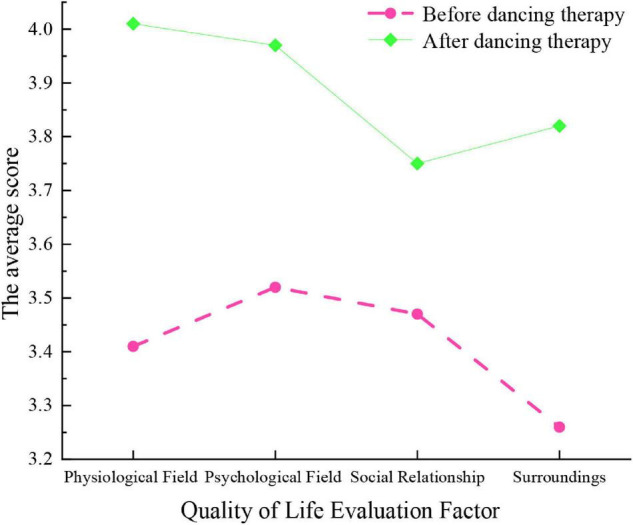
Scores of college students’ life quality before and after DT.

The test results of college students’ life quality in the group before and after DT are shown in Figure 5.

Figure 5 reveals that after the DT intervention, the QS results of college students’ life quality have significantly improved from physiology, psychology, environment, and social relations, proving that DT enhances college students’ life quality in all aspects. The data results illustrate the superiority and influence of DT in CSLE and deepen the understanding and research on their relationship. This is conducive to supporting relevant education programs of the combination of DT and CSLE (Hyvönen et al., 2020).

**FIGURE 6:**
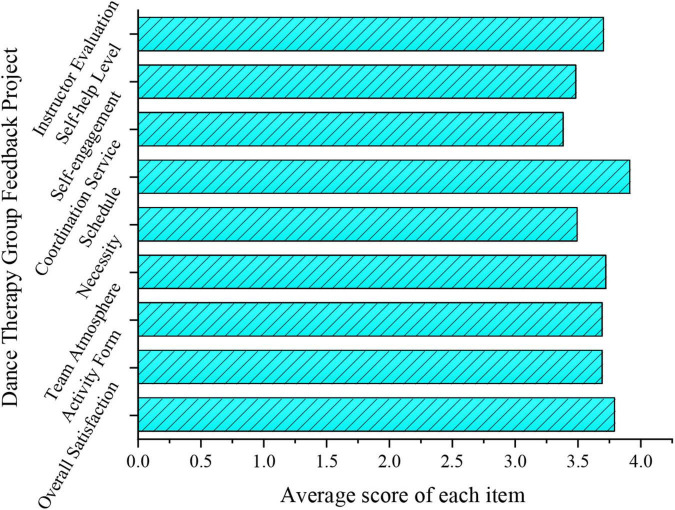
Average scores of the group counseling project for college students after DT.

After all the activities of the experimental group, the subjects are asked to fill in the scoring results of the group counseling feedback form, as shown in Figure 6.

Figure 6 presents the scoring results of the feedback table. Clearly, the deviation of several items of the group counseling project is not very large, and the coordination service is more evident because of members’ personal problems, which is not the main effect of DT. The scores of self-engagement and self-help level prove the positive role of DT in CSLE (Qian et al., 2018).

**FIGURE 7:**
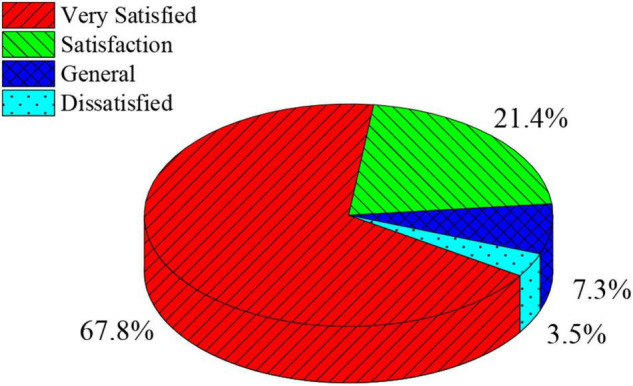
QS on college students’ satisfaction with group counseling after dT.

The QS results of group members’ satisfaction with DT are shown in Figure 7.

Figure 7 suggests that after the DT, the satisfaction QS results of the group members show that most members are delighted with DT activities, and only 3.5% are dissatisfied with all DT activities. Thus, the influence of DT is proved on CSLE.

In summary, DT has improved college students’ life values and life quality. DT is very effective at all levels of CSLE (Jennifer, 2020), which is detailed below: (1) DT has enhanced students’ understanding of the object. The group data analysis results of DT imply that the improvement in the physiological dimension of college students’ life quality after DT activities is undeniable; that is, DT is very effective in understanding life at the body level so that college students have more explicit attention and understanding of their own existence. (2) DT can raise students’ life consciousness. The group DT activities have a good intervention and transformation on the negative emotions of college students and inspire them to maintain a positive attitude toward life. Thus, DT improves college students’ life consciousness. (3) DT improves students’ interpersonal relationships. In the DT group activities, activity scope, and modes of dance movements are adjusted to train college students to experience the influence of interpersonal relationships in these activities. Meanwhile, through targeted exercise for students’ interpersonal skills, students can feel the group’s strength and establish a complete self-boundary, enhancing their interpersonal communication skills. In general, DT in life education has significantly improved the subject and object cognition, consciousness, and interpersonal relationship of college students, which confirms the effectiveness of DT in CSLE. Moreover, DT can also significantly promote the IAPE closely related to CSLE (Kita, 2020; Raybin and Krajicek, 2020).

### Analysis of Educational Psychology in Life Education

**FIGURE 8:**
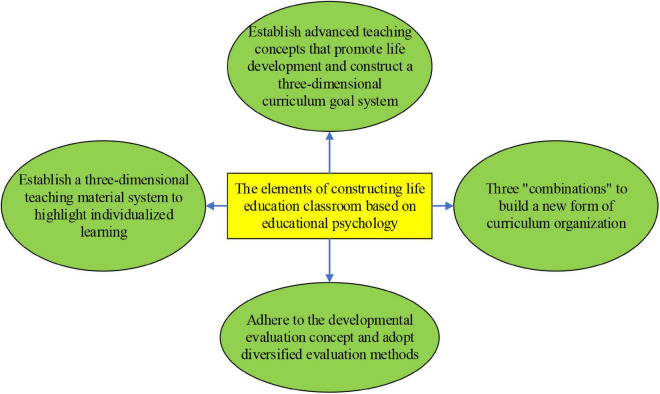
Elements of CSLE curriculum based on EP.

The construction of the life education classroom under EP is outlined in Figure 8 (Priniski et al., 2019).

As shown in Figure 8, the impact of EP on the construction of the CSLE curriculum is summarized from four aspects. The CSLE curriculum under EP is deeply analyzed and studied from these aspects. The result analysis indicates that the curriculum based on EP raises the influence of CSLE (Garvey et al., 2019; Mancini et al., 2020). The contents of these four aspects are as follows: (1) Establish a teaching concept for promoting life development and construction of a 3D (Three-Dimensional) curriculum objective system. According to the core concept of EP, CSLE has become more student-centered, and the goal of the new curriculum focuses on human development, breaking through the single cognitive goal, emphasizing autonomous learning ability, and encouraging students to participate in social activities actively. (2) Establish a 3D teaching material system that highlights personalized learning. CSLE respects individuals’ uniqueness, stimulates students’ potential, and helps them develop with the most suitable approach based on their personalities. (3) Construct a new curriculum organization based on three “combinations.” The development of EP should follow the transitions of the era. Thus, there is a need to combine curriculum reform of EP with the new network technology, theory and object research, content learning, and social practice, thereby stimulating students’ learning motivation, innovative learning abilities, cooperative spirits, and theoretical techniques (Ashleigh et al., 2019). (4) Develop evaluation concepts and diversify evaluation methods. EP has dramatically changed the life education curriculum and the evaluation method of the curriculum. The developmental evaluation concept is helpful to master the growth and progress of students. The evaluation method on CSLE under EP has greatly stimulated students’ learning enthusiasm and promotes CSLE (Rogoza et al., 2018; Michael, 2019).

### Analysis of Educational Psychology-Based Dance Therapy in College Students’ Life Education

This section analyzes applying DT in CSLE under EP and the effect of DT on CSLE. Although there are still some problems with DT and CSLE in China, the research on their future development is promising. Consequently, in-depth analysis and discussion are conducted on the relationship between CSLE and DT (Janet, 2019).

Dance therapy focuses the consciousness on the body through the activities and perception of the body to have more precise attention and understanding of their own object life. Meanwhile, DT focuses on the less active parts in life so that all aspects of the body can be coordinated and unified to carry life better. Additionally, DT requires the subjects to do semi-free warm-up activities to freely feel their own body and the energy flow inside the body. By doing so, the subjects can actively focus their consciousness on the body itself and transfer their attention from the outer-self to the inner-self. Then, they will have an insight into their body from the outside to the knowledge. By using the limbs to express freely with the music, the subjects can express their emotions freely because the concealment is good in the expression process, making the subjects more willing to use the limbs to tell their inner world and even their concealed emotions. The leader will also pay attention to everyone’s free expression and mirror it. The subject can affirm external limb movements, strengthen his enthusiasm, explore themselves more autonomously, and get twice the result with half the effort.

Dance therapy can improve college students’ outlook on life. Because in the activity design process, the negative emotions are well intervened and transformed while the subjects are intervened to remain in a positive life state to alter the outlook on life to a more positive aspect. Apparently, DT is effective in improving college students’ consciousness and concept. During the activity, the subjects can feel their body emotion and express them through the body. Further, they can discover their uncomfortable or unsmooth body parts when feeling this emotion and adjust the body through some stretching actions to regulate emotion. Such effects are also involved in psychoanalysis and psychodynamic theory: through energy dredging, energy injection, and other activities, the positive state is injected into the body utilizing intention, suggestion, and metaphor, to improve the subjects’ understanding of life and positive attitude.

Dance therapy has improved the interpersonal relationship in CSLE. In the group activity design of DT, students can experience the impact of different actions on interpersonal relationships by training and adjusting the scope and mode of action. At the same time, in group activities, students can effectively improve their interpersonal skills by purposefully exercising interpersonal skills, feeling group power and support, and establishing self-boundaries. Moreover, DT is effective in three main aspects of CSLE: understanding of object life, life view, and interpersonal relationship.

**FIGURE 9:**
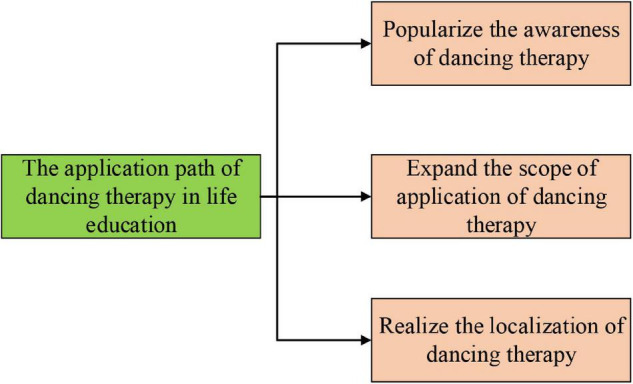
Path to popularize the application of DT in life education.

The application path analysis of DT in CSLE is shown in Figure 9 (Roswiyani et al., 2019; Saumaa, 2019).

As shown in Figure 9, DT, as a newly introduced art therapy method in China, should be given more opportunities and promotion paths in CSLE (Ruiz-Muelle and López-Rodríguez, 2019). Here, the following three points are summarized: (1) There is a need to popularize the understanding of DT among the public about its forms, benefits, and connotation, as well as the core of DT and psychotherapy by movement. (2) It benefits the public to expand the application of DT. With the increasing attention from the government and the mass to psychotherapy, the combination of DT with various fields is deepening, extending the scope of DT applications. (3) It is necessary to localize DT. After introducing DT into China, there has been a rising demand to create DT methods with Chinese characteristics by integrating China’s existing situation and cultural inheritance (Sliver, 2020).

The practical research results of DT in CSLE are shown in Table 2. It confirms the effectiveness of DT in life education. DT also provides practical and effective scheme research for CSLE. Teaching and learning should be implemented in the following aspects to develop CSLE (Stephen et al., 2020).

**TABLE 2:** Direction of CSLE.

Educational direction	Values consciousness	Methods of life education	Establishment of the education system
Content	There is a need for higher institutions to pay more attention to the CSLE. Relevant education enables college students to revise their life values from the ideological level to fundamentally solve the problems of weak life consciousness and confusion of life values for some college students	Combining CSLE and EP has produced many innovative educational approaches. With the continuous development of psychology and life education, there is a need to reform the educational methods	The syllabus and teaching materials of CSLE have not formed an integral and systemic structure. Most people who pay attention to CSLE are psychological teachers or counselors, while qualified teaching faculties are lacking. Such gap must be filled by establishing a complete life education system

As shown in Table 2, there are several vital directions in CSLE, in which DT can be used to solve the problems of life educational methods. In terms of values, DT can significantly improve the cultivation of college students’ life values, remedy their life value deviations through body movements, and enhance the consciousness of life values (Ndofirepi and Rambe, 2018; Seron, 2021). In terms of educational methods, DT is a highly interdisciplinary subject involving the fields of biology, neurology, and sociology. The life education methods of college students can be improved and adjusted from all aspects. Finally, the intersection of DT in psychotherapy accelerates establishing the CSLE system and promotes the compilation of CSLE textbooks (Carole, 2019).

Further, as the basis of CSLE, IAPE has broadened the life educational development through the combination with DT. In particular, the IAPE related to CSLE can be implemented by introducing the metaphors and hints in DT. Then, these contents are collectively combined with the mature IAPE system, thereby fully exerting the role of life education and diffusing the ideas of IAPE. Moreover, DT is a new way of education, enriching IAPE methods in higher institutions. These two subjects can be well-combined from multiple perspectives, such as students’ world outlook, values, and CSLE, thus gaining optimal results from the CSLE (Jean and Tara, 2020).

This manuscript studies the combination of DL technology and ER, which will impact the results of DT, realize the possibility of combining science and technology with art, and apply it to CSLE to make CSLE more efficient under the influence of new technology. Ye et al. (2021) combined DL technology with ER and gained detailed emotional feedback (Ye et al., 2021). Abbaschian et al. (2021) applied DL and ER in the classroom and achieved good results (Abbaschian et al., 2021). This manuscript uses DL technology to the final effect of DT and also has achieved good results. Specifically, the research findings provide a reference for the academic research of DL technology in DT and life education.

## Discussion

The experimental statistical results corroborate that after the intervention of the DT group counseling experimental group, all dimensions of the college students’ Life Values Scale have been significantly improved. Especially, self-development and dedication have risen from the second to the first and the fifth to the fourth, respectively. Thus, the DT group has a particular guiding role in college student’s life values. Carr et al. (2021) found that DT positively improved mental state by constructing a DT model (Carr et al., 2021). After the intervention, the DT group counseling experimental group scores in the physical field, psychological field, social relationship field, and environmental field of the Life Quality Scale have significantly improved. Hence, group DT is helpful to enhance all aspects of college student’s life quality. The results are consistent with the study of Pylvänäinen et al. (2020), who believed that DT could reduce people’s depression index and improve the quality of life (Pylvänäinen et al., 2020). After the eight experimental activities, the respondents fill in the feedback form. The results show that all team members are very satisfied with the DT group counseling and think it is gratifying. The average score of all respondents in the feedback form is 3.66, and the respondents powerfully affirm the effect of DT group counseling. The latest research of Bendel-Rozow (2021) uncovered an excellent result of the group effect of DT in treating severe depression (Bendel-Rozow, 2021), which is consistent with the research conclusion of this study manuscript. In terms of several items of DT group counseling itself, there is a large deviation from the overall average value of 3.66 compared with other items in terms of coordination service and time arrangement.

## Conclusion

The present work introduces the relevant theoretical concepts of EP, life education, and DL. It also analyzes the relationship between DL and CSLE and the experimental data of DT on college students’ life values and life quality. Finally, it discusses the task and function of life education theory based on EP in CSLE. The results show that the EP-based life education theory can well promote CSLE and play a positive role in the development of CSLE. Meanwhile, DT can encourage people to focus on life education and help improve the CSLE system in China. With the application of EP, students’ acceptance of knowledge is higher, promoting the research and development of EP. The application of DT based on DL and EP in CSLE provides new ideas and schemes for CSLE, innovates the achievements of EP and DT in life education, provides a theoretical and data basis for future research, and promotes the development of CSLE. The deficiency is that DT and CSLE development in China is relatively short, and there are still some problems, so the research is not deep enough. For example, there are a few items in the QS, and the presentation effect of the DT is not detailed enough. Therefore, the follow-up research will deeply study the role of dance learning and psychological ideas in CSLE to get more specific research results. The results fully show that DT has more possibilities and broader development prospects in CSLE. The research provides a reference for developing DL technology in the psychology field. The conclusion provides support for the further promotion of DT in higher institutions. Also, it provides theoretical and experimental data support for the vital role of sports in the field of psychology.

## Data Availability Statement

The raw data supporting the conclusions of this article will be made available by the authors, without undue reservation.

## Ethics Statement

The studies involving human participants were reviewed and approved by Ethics Committee of Northeast Agricultural University. The patients/participants provided their written informed consent to participate in this study. Written informed consent was obtained from the individual(s) for the publication of any potentially identifiable images or data included in this article.

## Author Contributions

All authors listed have made a substantial, direct, and intellectual contribution to the work, and approved it for publication.

## Conflict of Interest

The authors declare that the research was conducted in the absence of any commercial or financial relationships that could be construed as a potential conflict of interest.

## Publisher’s Note

All claims expressed in this article are solely those of the authors and do not necessarily represent those of their affiliated organizations, or those of the publisher, the editors and the reviewers. Any product that may be evaluated in this article, or claim that may be made by its manufacturer, is not guaranteed or endorsed by the publisher.

## References

[B1] Abbaschian B. J. Sierra-Sosa D. Elmaghraby A. (2021). Deep learning techniques for speech emotion recognition, from databases to models. *Sensors* 21:1249. 10.3390/S21041249 33578714 PMC7916477

[B2] Abdullah S. M. S. A. Ameen S. Y. A. Sadeeq M. A. Zeebaree S. (2021). Multimodal emotion recognition using deep learning. *J. Appl. Sci. Technol. Trends* 2 52–58. 10.1088/1742-6596/1916/1/012118

[B3] Allen K. R. Lavender-Stott E. S. (2020). Preparing the educators who teach about families: engaging family science in the university setting. *Family Relations* 69 442–460. 10.1111/fare.12421

[B4] Ashleigh H. Joseph R. Shawn M. D. Abigail B. (2019). Peer mentoring to prepare high school students with autism spectrum disorder for college. *AND* 3 411–422. 10.1007/s41252-019-00132-y

[B5] Aziz Y. Khan A. Y. Shahid I. Khan M. A. Aisha. (2020). Quality of life of students of a private medical college. *Pak. J. Med. Sci.* 36 255–259. 10.12669/pjms.36.2.668 32063970 PMC6994898

[B6] Bendel-Rozow T. (2021). Recovery-Oriented dance movement therapy group with adults coping with severe mental health conditions: a controlled trial. *Arts Psychotherapy* 75:101830. 10.1016/j.aip.2021.101830

[B7] Brotherson S. E. Hoffman M. S. (2020). The history and usage of parenting newsletter interventions in family life education. *Educ. Sci.* 10 326–326. 10.3390/educsci10110326

[B8] Burchell A. (2021). At the margins of the medical? Educational psychology, child guidance and therapy in provincial England, c.1945–74. *SHM* 34 70–93. 10.1093/SHM/HKZ097 33854409 PMC8025347

[B9] Candelieri I. (2020). Sound. *Gestalt Theory* 42 233–242. 10.2478/GTH-2020-2020

[B10] Cao W. Liu M. Kong S. Wu M. Zhang Y. Yang P. (2021). Recent advances in software tools for more generic and precise intact glycopeptide analysis. *Mol. Cell. Proteomics* 20:3721. 10.1074/mcp.R120.002090 33556625 PMC8724820

[B11] Carole K. (2019). Shamanism in weimar dance: the pathway to mary wigman and the beginning of dance as therapy. *Dance Res.* 37 165–180. 10.3366/drs.2019.0271

[B12] Carr C. Feldtkeller B. French J. Havsteen-Franklin D. Huet V. Karkou V. (2021). What makes us the same? what makes us different? development of a shared model and manual of group therapy practice across art therapy, dance movement therapy, and music therapy within community mental health care. *Arts Psychotherapy* 72:101747. 10.1016/j.aip.2020.101747

[B13] Chen M. (2019). The impact of expatriates’ cross-cultural adjustment on work stress and job involvement in the high-tech industry. *Front. Psychol.* 10:2228. 10.3389/fpsyg.2019.02228 31649581 PMC6794360

[B14] Clynes M. Sheridan A. Frazer K. (2020). Student engagement in higher education: a cross-sectional study of nursing students’ participation in college-based education in the Republic of Ireland. *Nurs. Deuc. Today* 93:104529. 10.1016/j.nedt.2020.104529 32663634

[B15] Gail S. Michal A. Y. (2020). Culturally sensitive dance movement therapy for ultra-orthodox women: group protocol targeting bodily and psychological self-perceptions. *Arts Psychotherapy* 71:101709. 10.1016/J.AIP.2020.101709

[B16] Garvey J. C. Viray S. Stango K. Estep C. Jaeger J. (2019). The emergence of third spaces: exploring trans students’ campus climate perceptions within collegiate environments. *Soc. Educ.* 92 229–246. 10.1177/0038040719839100

[B17] Gehlbach H. Robinson C. D. (2021). From old school to open science: the implications of new research norms for educational psychology and beyond. *Educ. Psychol.* 56 79–89. 10.1080/00461520.2021.1898961

[B18] HyeRyeon K. ChangHwan C. Eunhye J. (2020). A methodological quality assessment of meta-analysis studies in dance therapy using AMSTAR and AMSTAR 2. *Health Care* 8 446–446. 10.3390/healthcare8040446 33139623 PMC7711445

[B19] Hyvönen K. Pylvänäinen P. Muotka J. Lappalainen R. (2020). The effects of dance movement therapy in the treatment of depression: a multicenter, randomized controlled trial in finland. *Front. Psychol.* 11:1687. 10.3389/fpsyg.2020.01687 32903394 PMC7434972

[B20] Janet L. W. (2019). (Re-) defining dance/movement therapy fifty years hence. *Am. J. Dance Therapy* 41 273–285. 10.1007/s10465-019-09295-9296

[B21] Jean R. Tara S. (2020). The shady pink elephant: end of life education for young women affected by Breast Cancer. *J. Cancer Educ.* 35 100–104. 10.1007/s13187-018-1446-1441 30591991 PMC6971143

[B22] Jennifer F. T. (2020). Abstracts from the 2019 Research and Thesis Poster Session of the 54th Annual American Dance Therapy Association Conference, Miami, Florida. *Am. J. Dance Therapy* 42 107–126. 10.1007/s10465-020-09322-x

[B23] Kita E. (2020). With compass and plumb-line: a dance movement therapy systemic approach in the field of the refugee crisis. *Body Mov. Dance Psych.* 15 171–188. 10.1080/17432979.2020.1778529

[B24] Ko K. S. (2020). East Asian dance/movement therapy educators’ experiences of teaching dance/movement therapy in East Asia after training in the US. *Arts Psychotherapy* 71:101711. 10.1016/J.AIP.2020.101711

[B25] Koch S. C. Riege R. F. Tisborn K. Biondo J. Martin L. Beelmann A. (2019). Effects of dance movement therapy and dance on health-related psychological outcomes. a meta-analysis update. *Front. Psychol.* 10:1806. 10.3389/fpsyg.2019.01806 31481910 PMC6710484

[B26] Mancini J. A. O’Neal C. W. Lucier-Greer M. (2020). Toward a framework for military family life education: culture, context, content, and practice. *Family Relations* 69 644–661. 10.1111/fare.12426

[B27] Michael L. (2019). Making sense of “Outsiderness”: how life history informs the college experiences of “Nontraditional” students. *Qual. Inquiry* 25 500–512. 10.1177/1077800418817839

[B28] Millman L. M. Terhune D. B. Hunter E. C. Orgs G. (2021). Towards a neurocognitive approach to dance movement therapy for mental health: a systematic review. *Clin. Psychol. Psychotherapy* 28 24–38. 10.1002/cpp.2490 32539160

[B29] Morris M. E. Slade S. C. Wittwer J. E. Blackberry I. Haines S. Hackney M. E. (2021). Online dance therapy for people with Parkinson’s disease: feasibility and impact on consumer engagement. *Neurorehabil. Neural Repair* 45:15459683211046254. 10.1177/15459683211046254 34587834

[B30] Ndofirepi T. M. Rambe P. (2018). A qualitative approach to the entrepreneurial education and intentions nexus: a case of Zimbabwean polytechnic students. *SAJESBM* 10 e1–e14. 10.4102/sajesbm.v10i1.81

[B31] Priniski S. J. Rosenzweig E. Q. Canning E. A. Hecht C. A. Tibbetts Y. Hyde J. S. (2019). The benefits of combining value for the self and others in utility-value interventions. *J. Educ. Psychol.* 111:1478. 10.1037/edu0000343 31772414 PMC6879189

[B32] Pylvänäinen P. Hyvönen K. Muotka J. (2020). The profiles of body image associate with changes in depression among participants in dance movement therapy group. *Front. Psychol.* 11:564788. 10.3389/fpsyg.2020.564788 33123046 PMC7573211

[B33] Qian J. Song B. Jin Z. Wang B. Chen H. (2018). Linking empowering leadership to task performance, taking charge, and voice: the mediating role of feedback-seeking. *Front. Psychol.* 9:2025. 10.3389/fpsyg.2018.02025 30410461 PMC6209672

[B34] Raybin J. L. Krajicek M. (2020). Creative arts therapy in the context of children with cancer: a concept analysis. *J. Pediatr. Oncol. Nurs.* 37 82–90. 10.1177/1043454219878397 31592707

[B35] Rogoza R. Żemojtel-Piotrowska M. Kwiatkowska M. M. Kwiatkowska K. (2018). The bright, the dark, and the blue face of narcissism: the Spectrum of Narcissism in its relations to the meta traits of personality, self-esteem, and the nomological network of shyness, loneliness, and empathy. *Front. Psychol.* 9:343. 10.3389/fpsyg.2018.00343 29593627 PMC5861199

[B36] Roswiyani R. Kwakkenbos L. Spijker J. Witteman C. L. (2019). The effectiveness of combining visual art activities and physical exercise for older adults on well-being or quality of life and mood: a scoping review. *J. Appl. Gerontol.* 38 1784–1804. 10.1177/0733464817743332 31640495 PMC6820121

[B37] Ruiz-Muelle A. López-Rodríguez M. M. (2019). Dance for people with Alzheimer’s disease: a systematic review. *Curr. Alzheimer Res.* 16 919–933. 10.2174/1567205016666190725151614 31345149

[B38] Saumaa H. (2019). Dance therapeutics: movement as a path toward healing. *Alternative Complementary Therapies* 25 238–240. 10.1089/act.2019.29238.has

[B39] Sengupta M. Banerjee M. (2020). Effect of dance movement therapy on improving communication and body attitude of the persons with autism, an experimental approach. *Body Mov. Dance Psych.* 15 267–279. 10.1080/17432979.2020.1794961

[B40] Seron L. C. (2021). Learning from the unconscious: psychoanalytic approaches in educational psychology. *Educ. Psychol. Pract.* 37 234–234. 10.1080/02667363.2021.1911519

[B41] Sliver B. R. (2020). On the margins of college life: the experiences of racial and ethnic minority men in the extra curriculum. *J. Contemporary Ethnography* 49 147–175. 10.1177/0891241619869808

[B42] Stănescu M. Tomescu G. (2021). The relationship between dance and multiple intelligences of institutionalised children: a theoretical framework for applied research. *BRAIN. Broad Res. Artificial Intell. Neurosci.* 11 167–184. 10.18662/brain/11.4Sup1/163

[B43] Stephen T. R. Allen B. M. Meg D. B. Armin A. D. (2020). Innovation and integration of sexuality in family life education. *Family Relations* 69 595–613. 10.1111/fare.12462 34588714 PMC8478349

[B44] Swaine B. Poncet F. Lachance B. Proulx G. C. Bergeron V. Brousse É (2020). The effectiveness of dance therapy as an adjunct to rehabilitation of adults with a physical disability. *Front. Psychol.* 11:1963. 10.3389/fpsyg.2020.01963 32982831 PMC7479122

[B45] Wei C. Yao H. Li D. (2021). Analysis of situation of life education for college students based on genetic simulated annealing algorithm. *Int. J. Early Childhood Special Educ. (INT-JECSE)* 30:385. 10.24205/03276716.2020.4037

[B46] Yamada M. Kawano T. (2021). Emerging wisdom through a traditional bon dance in group dance/movement therapy: a single case study of dementia. *Arts Psychotherapy* 75:101822. 10.1016/J.AIP.2021.101822

[B47] Ye Z. D. Pan D. Sun Z. Du C. G. Yin L. G. Long G. L. (2021). Generic security analysis framework for quantum secure direct communication. *Front. Phys.* 16:1–9. 10.1007/S11467-020-1025-X

