# Supplementary material for: Application of Educational Psychology-Based Dance Therapy in College Students’ Life Education
Source: Front Psychol. 2022 Mar 21;13:784568. doi: 10.3389/fpsyg.2022.784568 (PMC8977603; doi:10.3389/fpsyg.2022.784568)
Supplement: Supplementary file 1 [file Table_1.docx]

**Appendix**

**Appendix 1:**

QS on the application of DT in CSLE based on the concept of DP

The QS is divided into two parts and based on a four-point scoring system. Please score according to your own situation. This QS adopts an anonymous approach, and the results will be limited to academic research. Thank you for your full cooperation!

Group activity design and scoring

| Frequency | Unit name | Unit target | Scheduled activities | Duration | How satisfied are you |
| --- | --- | --- | --- | --- | --- |
| 1 | Hello everyone | Relationship building | Introduce treatment and establish a group contract | 1.5 hours |  |
| 2 | Do you understand your body? | Body perception | Feel your body | 1.5 hours |  |
| 3 | Body space exploration | Motion development | Practice in space | 1.5 hours |  |
| 4 | You, me, and him (her) | Relationship development | Feel yourself with others and groups | 1.5 hours |  |
| 5 | Body and emotion | Emotion expression | Build trust and express feelings with your body | 1.5 hours |  |
| 6 | Creative expression | Emotion transference | Release and transform destructive emotions and stimulate creative expression | 1.5 hours |  |
| 7 | Unity of body and mind | Body integration | eel the whole body and experience the entire body dialogue | 1.5 hours |  |
| 8 | Reborn | Group activity ending | Inject energy and hope into yourself, end the dance, group auxiliary feedback | 1.5 hours |  |

**Appendix 2:**

QS on the application of DT in CSLE based on the concept of Educational Psychology

This QS is divided into two parts with a four-point scoring system. Please score according to your situation. This QS adopts an anonymous system, and the results will be limited to academic research. Thank you for your full cooperation!

Group counseling design and scoring

| Times | Unit name | Unit objectives | Scheduled activity objectives | Activity content | Duration | How satisfied are you |
| --- | --- | --- | --- | --- | --- | --- |
| 1 | Hello everyone | Build relationships | Introduce treatment, establish a group contract, and initially try to use the body. | There are responsibilities introduction, self-introduction, warm-up activities, relationship establishment, and setting sail for the team. | 1.5 hours |  |
| 2 | Do you understand your body? | Body awareness | Feel your body | There are warm-up activities, feeling the body, experiencing the internal drive of action, combining internal drive, free expression, and end dance. | 1.5 hours |  |
| 3 | Explore body space | Motion development | Practice in space | There are warm-up activities, feeling the distance in different directions, feeling self space, feeling the space of others, and expressing freely. | 1.5 hours |  |
| 4 | You, me, and him | Relationship development | Feel yourself with others and groups | There are warm-up activities, "dancing scarves," end dance. | 1.5 hours |  |
| 5 | Body and emotion | Express emotions | Build trust and express emotions with your body | There are warm-up activities, feeling emotions, free expression, silk scarf sculpture. | 1.5 hours |  |
| 6 | Creative expression | Emotion transformation | Release and transform bad emotions and stimulate creative expression | There are warm-up activities, releasing emotions, transforming emotions, and expressing the end dance freely. | 1.5 hours |  |
| 7 | Unity of body and mind | Physical and mental integration | Feel the whole body and experience the whole body dialogue | There are warm-up activities, physical and mental integration, physical and mental dialogue, painting display, and free expression. | 1.5 hours |  |
| 8 | Reborn | Group end | energy and hope into yourself, end the dance, group auxiliary feedback | There are warm-up activities, end dance, perfect reply, happy car wash, end dance. | 1.5 hours |  |

Life education QS

Welcome to the survey on the life problems of college students. For each of the following questions, please choose an option that best suits your actual situation and real idea (1 = very inconsistent, 2 = relatively inconsistent, 3 = uncertain, 4 = relatively consistent, 5 = very consistent), and mark "√" on the corresponding number.

There is no right or wrong answer, and there is no need to overthink.

Note: Each question should be answered, and only one answer can be selected for each question. Please don't choose more or miss. Thank you for your cooperation!

| Content/Question item | Very inconsistent | Relatively inconsistent | Uncertain | Relatively consistent | Very consistent |
| --- | --- | --- | --- | --- | --- |
| 1. Only those who experience suffering will become complete people. | 1 | 2 | 3 | 4 | 5 |
| 2. If I die today, I will feel that my life is worthless. | 1 | 2 | 3 | 4 | 5 |
| 3. I think I am a contented person who lives in the present. | 1 | 2 | 3 | 4 | 5 |
| 4. Death is the end of life and has no other meaning. | 1 | 2 | 3 | 4 | 5 |
| 5. The course of life is to spend your life in a flat and light way. | 1 | 2 | 3 | 4 | 5 |
| 6. Suffering is a test of the strength of my character. | 1 | 2 | 3 | 4 | 5 |
| 7. I want to be a useful person to others, society, and the country. | 1 | 2 | 3 | 4 | 5 |
| 8. The life of experiencing suffering is the value of life. | 1 | 2 | 3 | 4 | 5 |
| 9. I appreciate the lifestyle of letting nature take its course and letting things go. | 1 | 2 | 3 | 4 | 5 |
| 10. What I do is experience setbacks and challenges. | 1 | 2 | 3 | 4 | 5 |
| 11. The fact that I will die one day makes me feel that my life is worthless. | 1 | 2 | 3 | 4 | 5 |
| 12. The real meaning of life is to experience suffering. | 1 | 2 | 3 | 4 | 5 |
| 13. If a person can realize his potential and give full play to his potential, his life is perfect. | 1 | 2 | 3 | 4 | 5 |
| 14. I want to be a person who serves the people like Lei Feng. | 1 | 2 | 3 | 4 | 5 |
| 15. Only when a person's potential is brought into full play and shown can he feel the greatest satisfaction. | 1 | 2 | 3 | 4 | 5 |
| 16. Tao Yuanming's description of a paradise free from worldly strife is the way of life I yearn for. | 1 | 2 | 3 | 4 | 5 |
| 17. If a person can be recognized and respected by others or society, he will realize the meaning of life. | 1 | 2 | 3 | 4 | 5 |
| 18. After suffering, I become more understanding of life. | 1 | 2 | 3 | 4 | 5 |
| 19. I appreciate people who can try their best to tap their potential and show their abilities. | 1 | 2 | 3 | 4 | 5 |
| 20. Whether to do something or not, I think most is whether it can bring me happiness. | 1 | 2 | 3 | 4 | 5 |
| 21. I like to do what others want me to do. | 1 | 2 | 3 | 4 | 5 |
| 22. The purpose of my study and work is to get happiness from it. | 1 | 2 | 3 | 4 | 5 |
| 23. When looking for a job, I pay more attention to whether it can give me a lot of room for growth and development, regardless of salary. | 1 | 2 | 3 | 4 | 5 |
| 24. If I can make the people around me happy and happy, I am willing to bear the pain alone. | 1 | 2 | 3 | 4 | 5 |
| 25. People are born to enjoy life. | 1 | 2 | 3 | 4 | 5 |
